# Functional Proteomics Screen Enables Enrichment of Distinct Cell Types from Human Pancreatic Islets

**DOI:** 10.1371/journal.pone.0115100

**Published:** 2015-02-23

**Authors:** Revital Sharivkin, Michael D. Walker, Yoav Soen

**Affiliations:** Department of Biological Chemistry, Weizmann Institute of Science, Rehovot, 76100, Israel; University of Lille Nord de France, FRANCE

## Abstract

The current world-wide epidemic of diabetes has prompted attempts to generate new sources of insulin-producing cells for cell replacement therapy. An inherent challenge in many of these strategies is the lack of cell-surface markers permitting isolation and characterization of specific cell types from differentiating stem cell populations. Here we introduce an iterative proteomics procedure allowing tag-free isolation of cell types based on their function. Our method detects and associates specific cell-surface markers with particular cell functionality by coupling cell capture on antibody arrays with immunofluorescent labeling. Using this approach in an iterative manner, we discovered marker combinations capable of enriching for discrete pancreatic cell subtypes from human islets of Langerhans: insulin-producing beta cells (CD9^high^/CD56^+^), glucagon-producing alpha cells (CD9^-^ /CD56^+^) and trypsin-producing acinar cells (CD9^-^ /CD56^-^). This strategy may assist future beta cell research and the development of diagnostic tools for diabetes. It can also be applied more generally for function-based purification of desired cell types from other limited and heterogeneous biological samples.

## Introduction

Pancreatic beta cells regulate metabolic homeostasis by controlled secretion of insulin; impaired beta cell function leads to persistently elevated levels of blood glucose, the hallmark of diabetes. Cell replacement therapy is considered a promising approach towards curing diabetes [1,2], but it is currently limited by a severe shortage of donor tissue. This has motivated approaches capable of *in vitro* generation of functional insulin-producing cells [3–6]. However, the lack of identified, cell type-specific surface markers is a major obstacle for isolation of relevant cells.

Although a number of cell-surface markers have been correlated with endocrine pancreas cells, these typically show limited selectivity for specific endocrine cell types [7,8]. While the transmembrane protein TMEM27 is selectively expressed in human beta cells, its extracellular domain is cleaved in these cells [9], and it is not clear whether antibodies to this protein can be used to purify beta cells by flow cytometry or otherwise [10]. Other methods of beta cell enrichment are based on genetic marking [11], Newport green dye labeling [12], elimination of duct cells [7] and generation of hybridoma-derived antibodies enriching for different endocrine and non-endocrine cell types [13]. None of these techniques, however, relies on beta cell-specific surface markers, and isolated cell populations currently exhibit an unknown degree of heterogeneity. The same lack of marker information applies to other endocrine subsets in human pancreas (alpha cells, delta cells etc.).

The difficulty of identifying cell type-selective surface markers in limited heterogeneous samples impedes research and medical applications in many other fields. This has motivated the use of various methods for identifying cell-surface markers. Traditional methods were based on flow cytometry [14–16], analysis of gene expression patterns [17,18] and immunostaining [19,20]. Higher throughput proteomics approaches used antibody arrays for profiling cell-surface markers [21] and for discriminating cell populations based on differentially expressed markers [22]. These platforms are very efficient for identifying surface markers in a population of cells, but are insufficient for revealing association of specific markers with a particular cell type. There is therefore a great need for robust screening procedures capable of identifying markers designating cells of desired type or function. Since tissue samples are often limited in quantity and availability, such procedures should permit functional analysis of multiple markers in parallel using small numbers of cells.

Here we introduce an iterative and robust high throughput screen capable of identifying and associating multiple cell-surface markers with functional cell-specific properties, such as insulin and glucagon production. The technique, termed Functional Cell Capture Screen (FCCS), allows highly efficient and rapid screening of cell type-specific markers in limited and heterogeneous samples. We demonstrate the efficiency and specificity of this approach by identifying novel markers enriching for beta, alpha, delta and acinar cells from cadaveric samples of human pancreatic islets of Langerhans. Importantly, the technique can be applied more generally for efficient identification of surface markers associated with cell sub-populations within limited and heterogeneous samples.

## Materials and Methods

### Ethics Statement

Human islets were provided through the European Consortium for Islet Transplantation (ECIT) for Basic Research program (JDRF award 31–2008–416). Experiments were approved by the Weizmann Institute of Science Bioethics and ESCRO Committee.

### Cell culture

Samples of islets cells were incubated for 48 hours in suspension (90 mm culture dish, Miniplast, Ein Shemer, 20090–01) in human islets medium: CMRL 1066 (Biological industries, 01–821–1A), 5.6mM glucose, 10% FBS (Biological Industries, 04–007–1A), 1% PEN-STREP-AMPHO (Biological Industries, 03–033–1B).

### Flow cytometry

Cells were dissociated using TrypLE Express (Invitrogen 12604) for 4 minutes, followed by quenching with 10% FBS in PBS. Blocking was performed in 10% FBS in PBS for 45 minutes on ice. Staining of cells was carried out in PBS containing 3% FBS using the following antibodies (BD biosciences): mouse anti-human CD44 (555476), mouse anti-human CD49B (555497), mouse anti-human EGFR (555996), mouse anti-human CD9 (555370), FITC mouse anti-human CD9 (312104), mouse anti-human CD56 (555514), APC mouse anti-human CD56 (555518), F(ab’)2 donkey anti-mouse PE (eBiosciences, 12–4012–87) and Alexa Fluor 647. Thresholds were determined using goat anti-mouse IgG1 k isotype control (eBiosciences, 14–4714–81) as follows: we defined gating that includes over 99% of the IgG control data and set the threshold to 1 log_10_ above the boundary of this gate. We used propidium iodide (Biotium, 40016) at 2μg/ml to mark dead cells. Suspended cells were filtered through 40 μm nylon strainer (BD Falcon), and analyzed/sorted by FACSAria flow cytometer (BD). Intracellular labeling for FACS analysis was performed immediately after the extracellular labeling procedure described above. Cells were fixed and permeabilized in Cytofix/Cytoperm solution (BD biosciences, 554722) for 1 hour on ice. All washes and subsequent incubations were carried out in Perm/Wash buffer (BD biosciences, 554723). We used guinea-pig anti-insulin antibody (DAKO, A0564) diluted 1:200, mouse anti-glucagon (Abcam, ab10988) diluted 1:200 and goat anti-somatostatin (Santa Cruz biotechnology, SC-7819) diluted 1:1000, for primary antibodies and Cy5 donkey anti-guinea-pig (Jackson ImmunoResearch, 706–175–148), Cy3 donkey anti-mouse (eBiosciences, 12–4012–87) and Cy3 donkey anti-goat (Jackson ImmunoResearch, 705–165–147) diluted 1:200 for secondary antibody.

### Array fabrication

Arrays were printed in a Microgrid printer with solid pins (Total array Systems, BioRobotics) on hydrogel coated slides (Full Moon Biosystems) using a panel of 231 monoclonal mouse anti-human antibodies (BD biosciences). The antibodies were printed at a concentration of 0.5 mg/ml in five spots, each using a single stamp and with 750 μm spacing. Following printing, the arrays were hydrated in a humidifier at 4°C for 48 hours, and then dried for 10 minutes at room temperature.

### The FCCS procedure

Cells were dissociated using TrypLE Express (Invitrogen, 12604) for 4 minutes, followed by quenching with 10% FBS in PBS. They were then seeded on the array at a total concentration of ~5*10^6^ cells/ml in 250–500μl of human islets medium, supplemented with 2μl of DNase (Ambion, 2U/μl). Prior to incubation of cells on the array, the printed area was blocked for 3 minutes with 1% BSA in PBS solution. The blocking solution was replaced by the cell suspension, and the arrays were incubated for 1 hour at 37°C. Excess cells were removed in a large volume of PBS and the arrays were fixed in 4% paraformaldehyde solution for 10 minutes. Cells on the array were permeabilized in 0.2% triton X-100 solution for 20 minutes, washed twice with PBS and blocked for 45 minutes in blocking buffer (2% FBS, 2% BSA, 50mM glycine in PBS). After blocking, arrays were washed twice with PBS and incubated for 2 hours at room temp in working buffer (1:10 diluted blocking buffer added 0.1% of triton X-100) containing the primary antibodies: guinea-pig anti-insulin (DAKO, A0564), rabbit anti-glucagon (DAKO, A0565) and goat anti-somatostatin (Santa Cruz biotechnology, SC-7819) antibodies. Primary antibodies were removed and the arrays were washed three times with working buffer. Then, secondary antibodies were added in working buffer for a 45 minutes incubation period at room temp: Cy5 donkey anti-guinea-pig (Jackson ImmunoResearch, 706–175–148), AlexaFluor 488 donkey anti-rabbit (Jackson ImmunoResearch, 711–545–152), Cy3 donkey anti-goat (Jackson ImmunoResearch, 705–165–147). After the incubation period, arrays were washed three times in working buffer and imaged using automated, high content fluorescence microscopy (IXmicro, MDC).

### Real-time quantitative PCR

RNA from sorted populations of cells was isolated using RNeasy MinElute Cleanup kit (Qiagen, 74204). DNA was eliminated using TURBO DNA-free kit (Ambion, AM1907) and the mRNA was converted to cDNA using high-capacity cDNA Reverse Transcription kit (Ambion, 4374967). Transcript levels were measured using real-time qPCR on a 7900HT Fast Real-Time PCR machine (Applied Biosystems) using Power SYBR green PCR master mix (Applied Biosystems). Primer sequences are detailed in Table S1 in S1 File. The levels of each gene was normalized using *RPLP0* endogenous control mRNA.

### Statistics

P-values of gene expression differences were computed using two-sample Paired t-test (one-tail) with equal variances. Number of repeats (n) represents biological replicates using samples derived from different donors.

## Results

### The functional cell-capture screen

**Fig 1:**
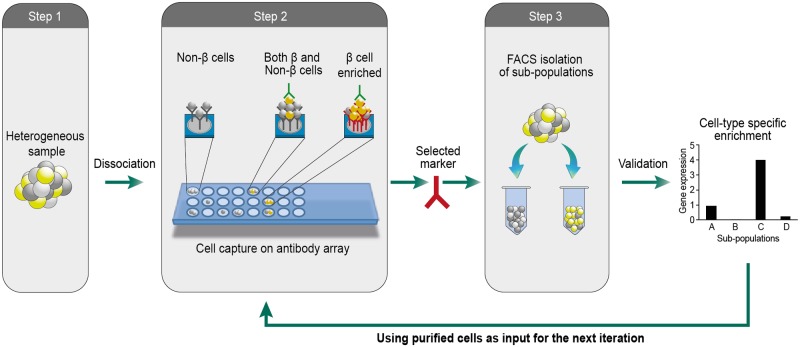
Schematic depiction of the FCCS approach. An array of antibodies against cell-surface antigens is printed on a hydrogel coated glass slide. Live cells in suspension are captured on specific antibody spots by interaction between their surface antigens and the printed antibodies. The captured cells are fixed and labeled for one or more intracellular markers of functional relevance (e.g. insulin, glucagon, somatostatin for pancreatic beta, alpha and delta cells, respectively). Populated spots enriched by cells with the desired label designate markers that are preferentially associated with the respective cell functionality. Following validation of the marker, it is used to isolate cells by flow cytometry. The isolated cells are then used as an input sample for the next iteration of this procedure. The iterative application of the procedure allows identification of additional markers that further refine the isolation.

We developed an iterative high throughput cell-capture screen for identification of cell-surface markers associated with cell type-specific functionality (Fig. 1). The procedure is performed in 3 steps which can be iterated to assemble marker combinations allowing progressive refinement of the isolation of desired cells. In the first step, we dissociate a heterogeneous sample into single cell suspension, and capture live cells on a hydrogel-coated glass slide printed with 231 different antibodies against cell-surface marker antigens (each antibody spot is represented in 5 replicates). Capture of cells on the array is based on recognition of cell-surface antigens by the printed antibodies. Each of the populated spots corresponds to an antigen expressed by a subset of cells which may contain one or more than one cell type. Identification of cell type-selective markers therefore requires mapping of identified antigens to specific cell types. To create this mapping we immunostain the cells tethered on the array with antibodies marking cell type-specific functionalities (Step 2). Analysis is performed by imaging the arrays with automated high content fluorescence microscopy (ImageXpress Micro), and calculating for each spot the fraction of cells positive for the relevant functional label. Spots enriched with positively-labeled cells define candidate surface markers for enrichment of cells with the desired functionality. In step 3, we FACS sort cells using these markers and validate cell type-specific enrichment by measuring expression levels of relevant functional genes. To further refine the enrichment, we repeat the entire 3-step procedure with cells that were pre-sorted using markers identified and validated in the preceding iterations.

### Screening for specific cell types within human islets of Langerhans

Islets of Langerhans from human pancreas provide a prominent example of clinically important tissue where research efforts are challenged by lack of efficient fractionation tools. The adult pancreas is composed of endocrine and exocrine cell populations. The endocrine cells are located within the islets of Langerhans, comprising cell types with discrete functionalities (e.g. alpha cells producing glucagon, beta cells producing insulin, delta cells producing somatostatin etc.) [23]. In addition to the heterogeneity of the islets themselves, islet preparations are often contaminated by varying fractions of exocrine tissue and even non-pancreatic cells. The exocrine tissue also comprises a number of different cell types, such as acinar cells which produce hydrolytic enzymes (e.g. trypsin, chymotrypsin, amylase and lipase) and duct cells [24].

**Fig 2:**
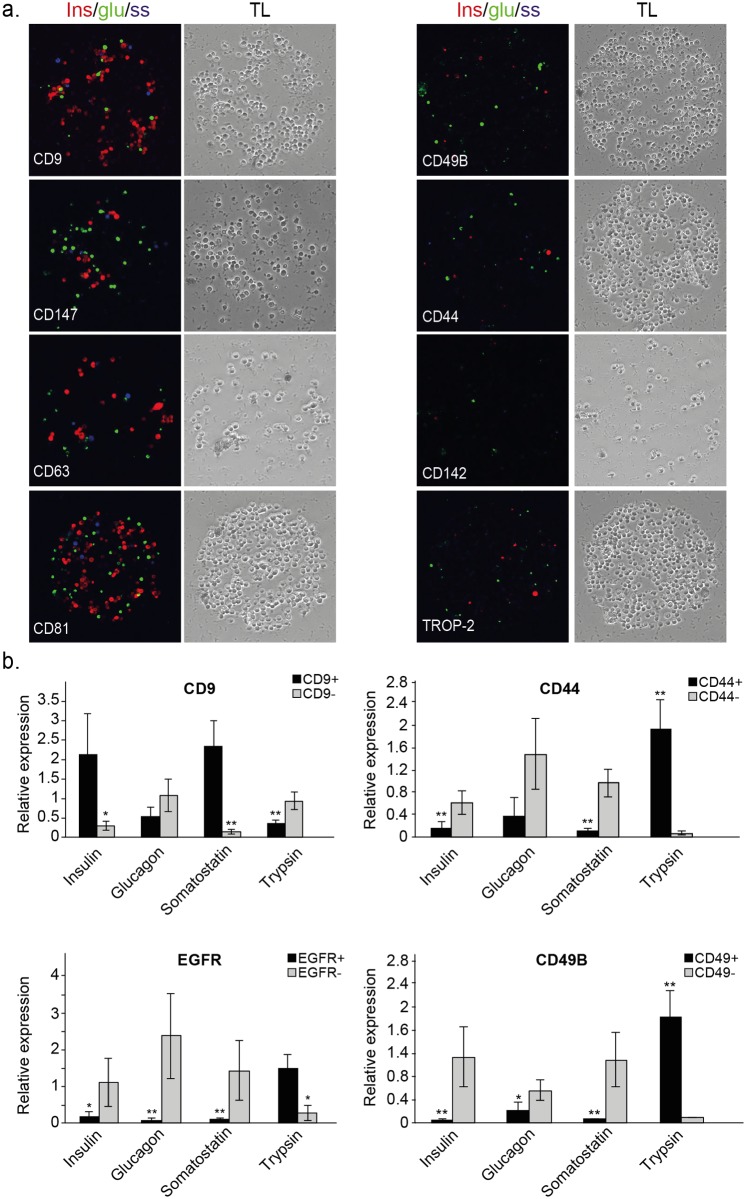
Identification and validation of markers for initial enrichment of specific cell types within human islets of Langerhans. **(a)** Representative images of populated antibody spots with different enrichments of insulin- (red), glucagon- (green) and somatostatin-positive cells (blue). On the right are spots mostly populated by non-endocrine cells, and on the left are spots populated by different proportions of alpha, beta and delta cells. Corresponding phase contrast images are shown on the right of each image (10x magnification). **(b)** Real-time qPCR analysis of several cell-type specific genes in different marker-isolated populations (insulin for beta cells, glucagon for alpha cells, somatostatin for delta cells and trypsin for acinar cells). CD9^+^ refers to top 10% expressing cells (CD9^high^). Shown is mean expression relative to unsorted (bulk) cells +/- SE (n = 3; biological replicates correspond to different donors; * p < 0.05, ** p < 0.01).

To identify and isolate specific cell types from islets of Langerhans, we obtained islets from pancreata of human cadavers. Analysis of cell capture on the antibody array, applied to samples from 3 different islet donors, identified 61 markers. Despite differences in donor age, gender, BMI and general condition of the islets, the majority of the markers were detected in at least two of the three donors (Table B in S1 File; Figure A in S1 File). Immunostaining of the captured cells for insulin, glucagon and somatostatin (Fig. 2a), revealed a heterogeneous labeling pattern: some of the populated spots, such as CD44 and CD142, were largely unstained, representing cell-surface markers that are mostly expressed by non-endocrine cells. Other spots (e.g. CD9, CD81 and CD147) contained a much higher percentage of labeled cells indicating more efficient capture of endocrine cells. Since none of the spots was populated exclusively by insulin, glucagon or somatostatin labeled cells, we used the newly identified markers as a starting point for a second iteration of the FCCS platform.

To prioritize markers for the second iteration, we examined the performance of several markers that were identified in the first iteration. For each marker, we isolated the respective cells by flow cytometry and compared the mRNA expression levels of insulin (beta cells), glucagon (alpha cells), somatostatin (delta cells) and trypsin (acinar cells) to the levels measured in negative cells. Some of these tested markers enriched for cells expressing higher levels of cell type-specific genes. In particular, cells expressing high levels of CD9 (top 10%) exhibited higher mRNA levels of insulin and somatostatin, indicating enrichment for beta and delta cells, respectively (Fig. 2b). In contrast, CD44^+^, CD49B^+^ and EGFR^+^ cells had elevated mRNA levels of trypsin, indicating enrichment for acinar cells (Fig. 2b). The inverse fractions of these populations also enriched for specific cell types, as indicated, for example, by elevated glucagon expression in EGFR^-^ cells, suggesting enrichment for alpha cells. These results demonstrate the efficiency of this functional platform for rapid identification of relevant marker combinations in a single experiment.

### Refining cell type-specific enrichment by a second iteration of FCCS

**Fig 3:**
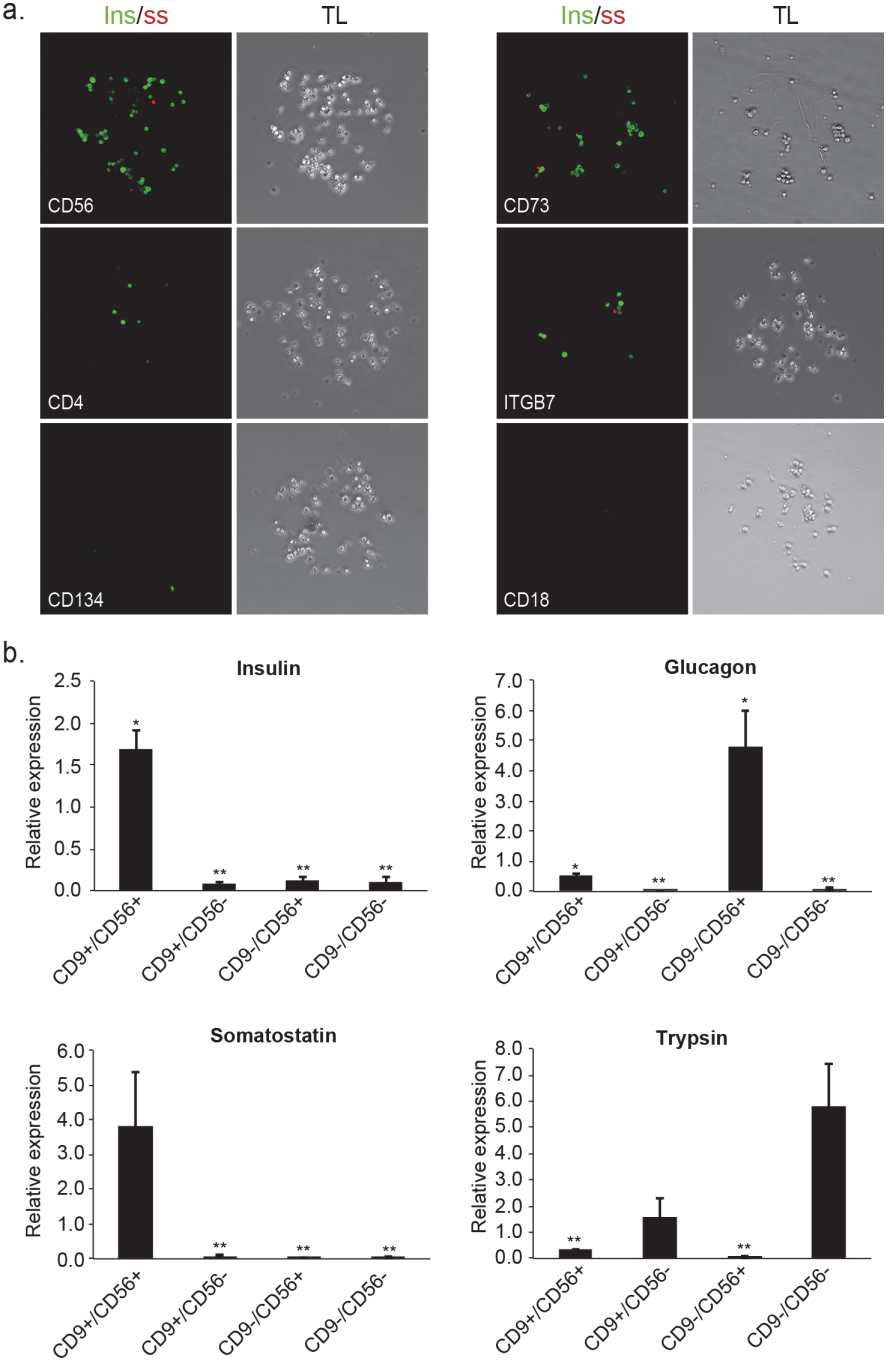
Second iteration of FCCS identifies marker combinations for improving the isolation of insulin producing cells. **(a)** Representative images of spots containing top 10% CD9-expressing cells (CD9^high^), immunostained for insulin and somatostatin. Spots with high, medium and low beta cell enrichment are presented from top to bottom. Respective phase contrast images (10x magnification) are shown to the right. **(b)** qPCR analysis of cell-type specific genes in islet cells fractionated based on CD9/CD56 combinations (CD9^high^/CD56^+^, CD9^high^/CD56^-^, CD9^-^/CD56^+^ and CD9^-^/CD56^-^). Shown is mean expression relative to unsorted (bulk) cells +/- SE (n = 3; biological replicates correspond to different donors; * p < 0.05, ** p < 0.01).

We refined the purification of beta cells by sorting CD9^high^ cells (top 10%), incubating them on the array and immunostaining the captured cells for insulin and somatostatin. The patterns of binding to the array identified 19 cell-surface antigens that were co-expressed with CD9 in two independent experiments (Table 1). Two of these markers, CD73 and CD56, corresponded to antibody spots that reproducibly enriched for cells exhibiting high levels of insulin staining (Fig. 3a). Despite the relatively low abundance of delta cells in islet samples, we also detected somatostatin positive cells in both CD73 and CD56 spots indicating co-enrichment of delta cells in addition to beta cells.

**Table 1:** Cell-surface markers expressed by CD9^**+**^ cells as detected by the second iteration of the FCCS.

Marker	Symbol	Avg % Ins^+^ cell	Avg % SS^+^ cell
CD4	CD4	10.3	0.3
CD73	NT5E	77.4	2.7
CD87	PLAUR	17.1	3.1
CCR4	CD194	20.0	4.9
CD165	AD2/APOE	25.8	1.1
CD85J	LILRB1	39.9	2.1
CD221	IGF1R	53.0	1.7
CD142	F3	12.6	1.9
CD134	TNFRSF4	6.0	0.0
ITGRN	ITGB7	16.9	0.9
CD68	CD68	11.2	0.3
CD56	NCAM1	59.5	2.1
WNT16	WNT16	48.7	3.4
CD18	ITGB2	9.6	0.7
CD6	CD6	37.7	1.0
CD77	A4GALT	44.2	1.2
CD61	ITGB3	49.6	1.0
CD32	FCGR2A	40.5	1.9

Displayed is the frequency of beta and delta cells in the total number of captured cells, averaged across the replicates of each spot.

We evaluated the CD9/CD56 and CD9/CD73 marker combinations by FACS sorting the respective cells and analyzing mRNA expression of cell type-specific genes. Notably, the fraction of CD9^high^/CD56^+^ cells was significantly larger than the CD9^high^/CD73^+^ fraction (Figure B in S1 File), indicating that CD56/CD9-based isolation can yield much larger numbers of cells. mRNA analysis of sorted CD9^high^/CD56^+^, CD9^high^/CD56^-^, CD9^-^/CD56^+^ and CD9^-^/CD56^-^ cells, revealed strong fraction-specific enrichments of different cell types. In particular, CD9^high^/CD56^+^ cells exhibited significantly higher levels of insulin and somatostatin compared with cells from all other fractions (Fig. 3b), indicating enrichment of beta and delta cells. This was further supported by insulin and somatostatin labeling of cells isolated using CD9 and CD56 (Figure C in S1 File). On the other hand, CD9^-^/CD56^+^ and CD9^-^/CD56^-^ cells exhibited much higher levels of glucagon and trypsin corresponding to enrichment of alpha and acinar cells, respectively (Fig. 3b).

**Fig 4:**
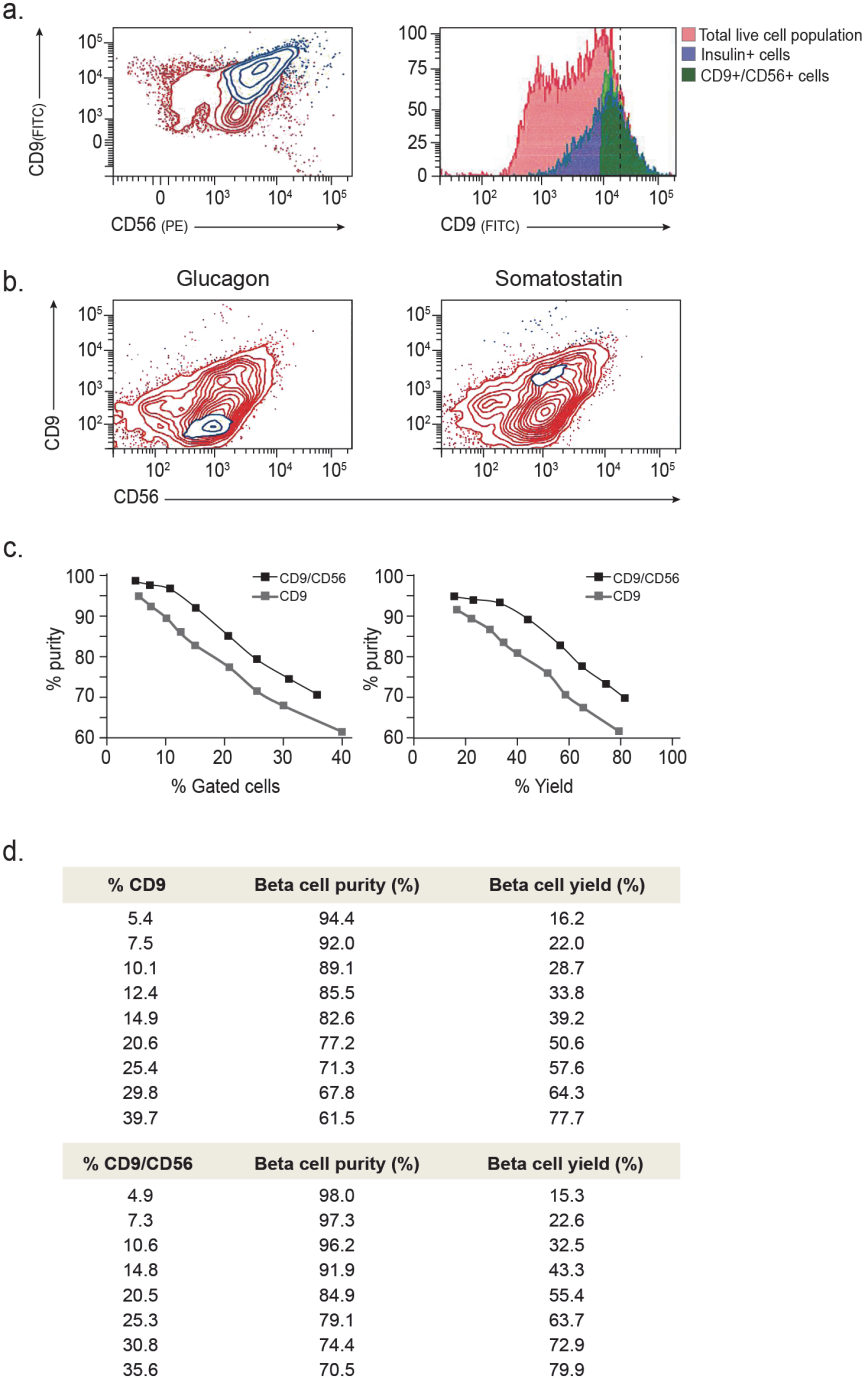
Improved purity and yield following a second iteration of the FCCS. **(a)** Left: Flow cytometry analysis of CD9, CD56 and intracellular insulin expression in islet samples. Co-localization of insulin^+^ cells (blue overlay) with CD9^high^/CD56^+^ expressing cells (determination of gated cells is described in S2 File and Figure D of S1 File). Insulin^-^ cells are shown in red. Right: histogram of all cells (red) vs. the insulin^+^ population (blue) and the top 20% of CD9-expressing cells within the CD56^+^ population (green). Dashed line indicates the gate for top 10% of the population by CD9 expression. This demonstrates the potential of the CD9/CD56 combination to identify more relevant cells as compared to only CD9. **(b)** Flow cytometry analysis of CD9, CD56 and intracellular glucagon (left) or somatostatin (right) in islets samples. Staining for glucagon and somatostatin is overlaid in blue on the CD9/CD56 panel, demonstrating co-localization of glucagon with CD9^-^/CD56^+^ expressing cells (left) and co-localization of somatostatin with CD9^high^/CD56^+^ (right). Glucagon and somatostatin negative cells are shown in red. **(c)** Left: comparison of purity (% of insulin^+^ cells in the isolated fraction) obtained by CD9/CD56-based isolation and CD9-based isolation at different choices of gating. Right: Comparison of CD9/CD56-based and CD9-based isolation with respect to beta cell yield (fraction of insulin^+^ cells out of total beta cells) and purity. The yield is calculated based on the same gates as the purity. Changes in CD9/CD56 gates were made by modifying the threshold of isolation equally along both axes. (**d**) Tabulation of the data points in (c).

Triple staining of islet cells with CD9, CD56 and insulin antibodies, followed by simultaneous intra- and extra-cellular FACS analysis, showed that insulin labeling was restricted to the CD9^high^/CD56^+^ compartment (Fig. 4a; Figure D in S1 File). Similar intra-cellular FACS analysis for glucagon and somatostatin (instead of insulin) revealed that alpha cells (glucagon^+^) localize to the CD9^-^/CD56^+^ compartment, while delta cells (somatostatin^+^) are restricted to the CD9^high^/CD56^+^ fraction (Fig. 4b).

To evaluate the added value of the second iteration, we compared the purity and yield of beta cells isolated based on the CD9/CD56 combination to isolation based on CD9 alone. We FACS analyzed the proportion of beta cells using a series of different gating options from very stringent to very permissive. While both single and double marker isolation schemes could achieve high cell purity, the CD9/CD56 based isolation outperformed the CD9-based isolation at all choices of gating (Fig. 4c). Increasing the fraction of sorted CD9 cells by lowering the CD9 expression threshold (isolating more than top 10%), considerably increased the fraction of non-beta cells. Much of this reduction in specificity was avoided when a similar-sized fraction was sorted using the CD9/CD56 combination. For example, CD9/CD56-based sorting of 10% of the total cell population resulted in 96% purity of beta cells compared to 89% purity based on CD9 alone (Fig. 4c). Thus, the use of the combined marker scheme allowed us to increase beta cell yield without compromising purity. This confirms that cell type-specific enrichment can be enhanced by iterating the FCCS procedure. Taken together, the results demonstrate the usefulness of this approach for reliable and efficient identification and isolation of specific cell types from a limited, heterogeneous and fluctuating cellular context.

## Discussion

Efficient utilization of human tissue and stem cells in basic and clinical applications will require improved methods for isolating and characterizing specific cell sub-populations from complex mixtures of cells. In this study, we used a novel proteomics approach to identify cell-surface marker combinations enabling high purity enrichment of several pancreatic cell subtypes, including insulin-producing cells. This approach can be used to enrich for cells with a desired function in a wide range of additional cellular contexts. It is especially advantageous for heterogeneous samples containing small numbers of cells and may currently be the only feasible way to perform a high-throughput screen in these cases.

Combining a functional readout with cell capture on the array represents a new and simple, yet powerful strategy to identify functionally relevant markers among the majority of other markers. This strategy solves two major problems simultaneously: 1) It enables screening of hundreds of cell-surface markers using small sample sizes (~4 x10^5^ cells), and 2) it reveals direct associations between cell-surface markers and desired cell-specific attributes (e.g. expression of insulin, somatostatin, glucagon, etc.). We have demonstrated the efficiency and specificity of the approach by identifying markers for purification of distinct pancreatic cell types within limited samples of human islets of Langerhans. This strategy was robust enough to yield reproducible results despite differences in donor age, gender and BMI which may dramatically affect sample composition.

Our analysis showed that different combinations of CD9 and CD56 enrich for different pancreatic cell types. CD9^high^/CD56^+^ enriched for beta and delta cells, CD9^-^/CD56^+^ for alpha cells and CD9^-^/CD56^-^ enriched for acinar cells.

Quantitative assessment of the purity and yield of isolated cells requires analysis of co-expression with lineage-specific genes at single cell resolution. We achieved this by combining intracellular FACS for insulin with standard FACS analysis of co-expression of either CD9 or CD9/CD56. Consistent with previous results [7], CD56 (or sialylated CD56), was not useful as a single marker for purification of insulin^+^ cells. Nevertheless, in combination with CD9, it facilitated efficient purification of beta cells. Moreover, the use of a combined, CD9/CD56 marker scheme outperformed the single marker isolations with respect to yield and purity. This highlights another important benefit of using FCCS, namely to identify marker combinations supporting increased purity without compromising the yield.

The enrichment of beta cells in the CD9^high^/CD56^+^ compartment was accompanied by enrichment of delta cells, despite their overall low abundance in islet samples. Delta cell contamination in beta cell preparations has been reported in previous isolations [13] and is consistent with the developmental proximity between these cell types. Indeed, the divergence of beta and delta cells is one of the last specification events of endocrine tissue in the embryo [25], perhaps leading to higher similarity between beta and delta cells as compared to other endocrine lineages. Discriminating beta from delta cells is therefore more difficult. In this regard, the capacity of the FCCS for parallel evaluation of multiple marker combinations in a single experiment, presents a major advance over other techniques. Hence, additional iterations of the FCCS may permit separation of beta and delta cells.

The ongoing world-wide epidemic of diabetes emphasizes the urgent need for improved understanding of endocrine pancreas development and beta cell biology. The CD9/CD56 marker combination identified in this study may contribute to this effort by providing an efficient means to enrich for mature beta cells using defined endogenous markers. The relevance of the newly identified markers (and their combinations) for beta cell precursors, or for beta cells derived from other sources, is currently unknown. However, the FCCS approach can be readily applied in these other contexts to identify combinations that are highly useful for the respective stage and cellular source.

While we demonstrated the usefulness of this strategy for human islet-based research, the approach could be extended to any cellular context for which a functional readout is available (e.g. labeled metabolites, granulation, ion content, mitotic state etc.). The FCCS may therefore constitute a general and efficient platform for resolving heterogeneous cellular systems.

## Supporting Information

S1 File Supporting tables and figures. **Table A**. Sequences of primers used in this study. **Table B**. Summary of results from 3 independent antibody array analyses of human islets of Langerhans, listing cell-surface markers detected in three donors. **Figure A**. Flow cytometry analysis of marker distribution in samples of human islets of Langerhans. Shown are distributions of markers that were detected by the antibody array in three different donors. Different markers display different expression patterns as determined by APC staining (blue) indicating that these markers can label several sub-populations of cells and may help resolve the heterogeneity of the sample. Cells that do not express the indicated marker are labeled red. Shown are live cells as determined by PI staining. **Figure B**. Expression patterns of marker combinations that enrich for insulin^+^ cells. Flow cytometry analysis of co-labeling of CD9 with CD56 or CD73. Color coding: red—low or no expression, purple—CD56 or CD73 positive cells, green and blue—cells with intermediate and high expression of CD9, respectively. Shown are live cells, determined by PI staining. **Figure C**. Enrichment of insulin^+^ and somatostatin^+^ cells in the CD9^+^/CD56^+^ compartment. Immunostaining for insulin (red) and somatostatin (green) in islet cells isolated based on different combinations of CD9 and CD56 expression (10x magnification). CD9^+^ refers to top 10% expressing cells (CD9^high^). **Figure D**. Determination of insulin^+^ cells. **(a)** Flow cytometry plot of islet cells stained for insulin. Insulin^+^ cells (blue) were determined based on IgG control. We defined a negative control gate containing over 99% of cells stained with IgG control and set a threshold for insulin^+^ cells at 1 log_10_ above the negative gate. The insulin axis is plotted against a non-specific fluorescence label (y-axis). **(b)** Distribution of the insulin^+^ cells (blue) with respect to the distribution of staining with CD9 and CD56. The same strategy of gating was used for the glucagon and somatostatin analysis of Fig. 4b. Additional information is provided in supporting experimental procedures within S2 File.
(DOCX)

S2 File Experimental Procedures. Procedure for combined extracellular and intracellular flow cytometry analysis.
(DOC)
