# Supplementary material for: Functional Proteomics Screen Enables Enrichment of Distinct Cell Types from Human Pancreatic Islets
Source: PLoS One. 2015 Feb 23;10(2):e0115100. doi: 10.1371/journal.pone.0115100 (PMC4338300; doi:10.1371/journal.pone.0115100)
Supplement: S1 File — Table A. Sequences of primers used in this study. Table B. Summary of results from 3 independent antibody array analyses of human islets of Langerhans, listing cell-surface markers detected in three donors. Figure A. Flow cytometry analysis of marker distribution in samples of human islets of Langerhans. Shown are distributions of markers that were detected by the antibody array in three different donors. Different markers display different expression patterns as determined by APC staining (blue) indicating that these markers can label several sub-populations of cells and may help resolve the heterogeneity of the sample. Cells that do not express the indicated marker are labeled red. Shown are live cells as determined by PI staining. Figure B. Expression patterns of marker combinations that enrich for insulin+ cells. Flow cytometry analysis of co-labeling of CD9 with CD56 or CD73. Color coding: red—low or no expression, purple—CD56 or CD73 positive cells, green and blue—cells with intermediate and high expression of CD9, respectively. Shown are live cells, determined by PI staining. Figure C. Enrichment of insulin+ and somatostatin+ cells in the CD9+/CD56+ compartment. Immunostaining for insulin (red) and somatostatin (green) in islet cells isolated based on different combinations of CD9 and CD56 expression (10x magnification). CD9+ refers to top 10% expressing cells (CD9high). Figure D. Determination of insulin+ cells. (a) Flow cytometry plot of islet cells stained for insulin. Insulin+ cells (blue) were determined based on IgG control. We defined a negative control gate containing over 99% of cells stained with IgG control and set a threshold for insulin+ cells at 1 log10 above the negative gate. The insulin axis is plotted against a non-specific fluorescence label (y-axis). (b) Distribution of the insulin+ cells (blue) with respect to the distribution of staining with CD9 and CD56. The same strategy of gating was used for the glucagon and somatostatin analysis of [file pone.0115100.s001.docx]

**SUPPORTING INFORMATION**

**Table A:** Sequences of primers used in this study.

| **Gene** | **Forward primer** | **Reverse primer** |
| --- | --- | --- |
| Insulin | GGGGAACGAGGCTTCTTCTAC | CACAATGCCACGCTTCTGG |
| Glucagon | GTGCAGTGGTTGATGAATACCAA | GTCTCTCAAATTCATCGTGACGTT |
| Somatostatin | CCCCAGACTCCGTCAGTTTC | CCGTCTGGTTGGGTTCAGA |
| Trypsin | GCTACAAGTCCCGCATCCA | TCCCCTCCAGGACTTCGAT |
| CD9 | CTGCCCCAAGAAGGACGTACT | CACTGCGCCGATGATGTG |
| EGFR | TCCAGTGGCGGGACATAGTC | TTTGGTCAGTTTCTGGCAGTTCT |
| CD44 | CCTTTGATGGACCAATTACCATAAC | TCAGGATTCGTTCTGTATTCTCCTT |
| CD49B | CAGGGCACTATCCGCACAA | TGTGACCAGAGTTGAACCACTTG |
| CD56 | GCCAACCCCACAGGAGTTC | AGCGATAAGTGCCCTCATCTG |
| RPLP0 | ACAGGGCGACCTGGAAGTC | TCTGCTCCCACAATGAAACATT |

**Table B:** Summary of results from 3 independent antibody array analyses of human islets of Langerhans, listing cell-surface markers detected in three donors.

| **Markers detected in 3 donors** | **Markers detected in 2 donors** | **Markers detected in 1 donor** |
| --- | --- | --- |
| CD147 | CD59 | CD221 3b7 |
| CD49F | CD55 | CLIP |
| CD42B | CD98 | CD28 |
| TROP-2 | BLD groupA | P-Glycoprotein |
| CD197 | CD53 | HLA-A2 |
| CD44 | CD29 | HMPT PRGN |
| CD49E | CD99 | CD66C |
| CD15 | CD61 | CLA |
| EGF R | C-ERB-2 | CD226 |
| CD71 | CD3 | CD66B |
| CD36 | β2-microglobulin | CD50 |
| CD177 | CD66 | CD55 |
| CD57 | CD90 | CDw93 |
| HLA-ABC | CXCR4 | LTBR |
| CD140B | CD54 | CD271 |
| CD142 | CD146 | CD45RB |
| CD81 |  | CD164 |
| CD63 |  | CD11B/MAC1 |
| CD49B |  | HLA-DR |
| KS1/4 |  | CD282 |
| CD47 |  | CD97 |
| CD9 |  | CD11C |
|  |  | CD166 |


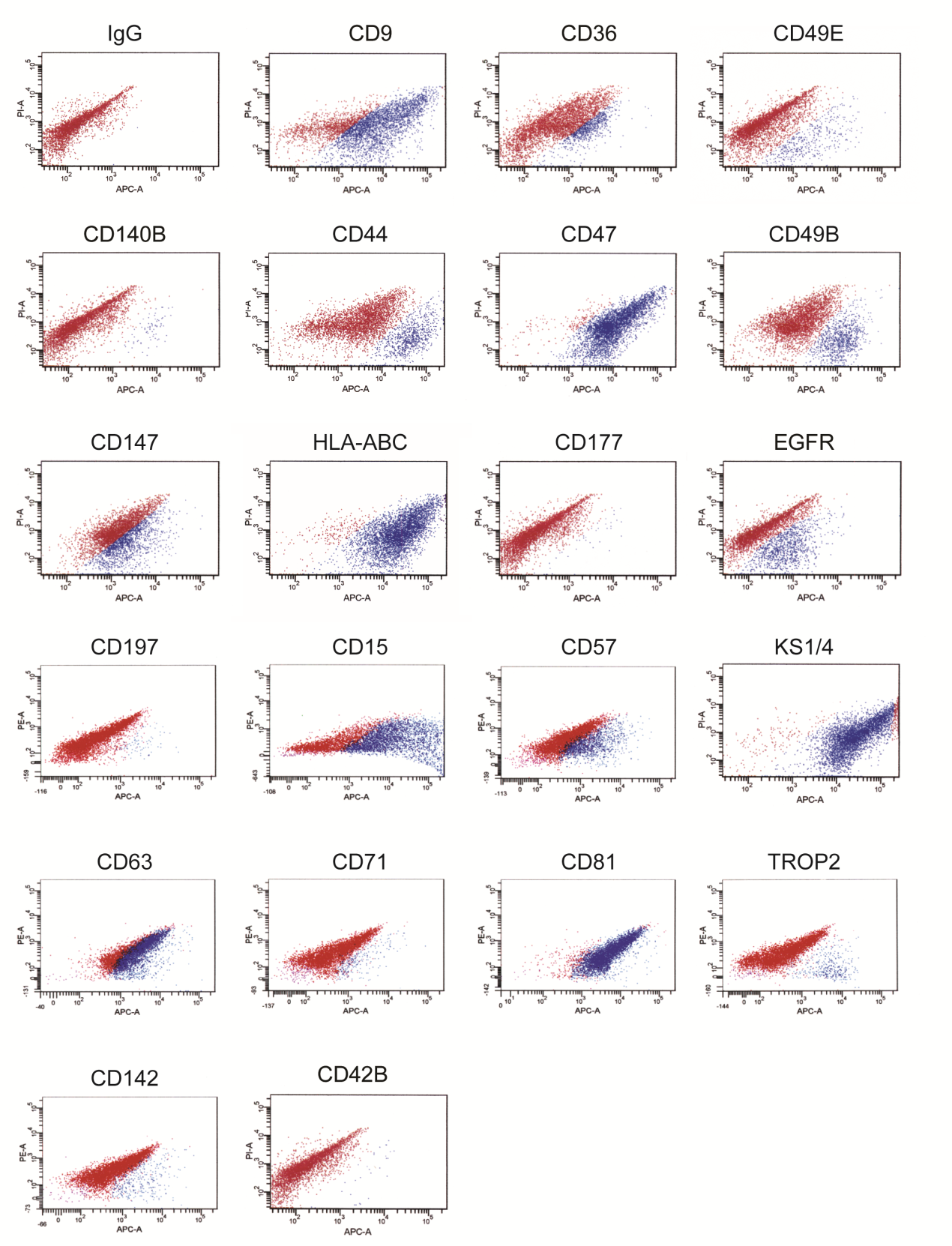


CD49F

CD49F


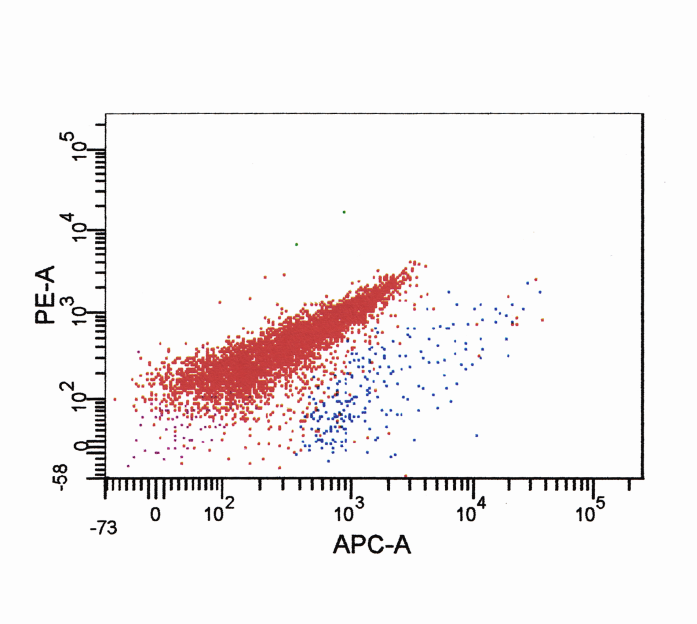


**Figure A: *Flow cytometry analysis of marker distribution in samples of human islets of Langerhans.***


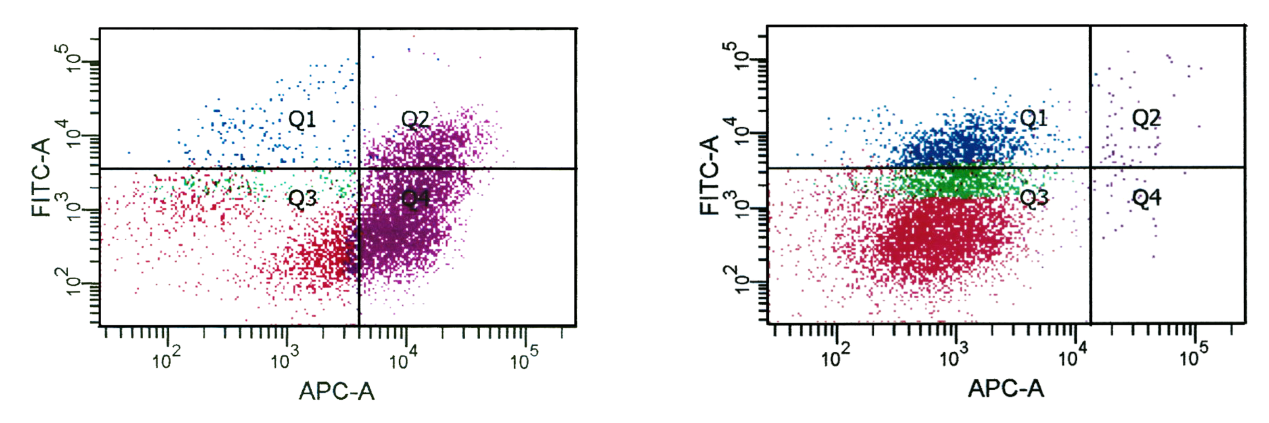


CD9

CD73

CD56

**Figure B: *Expression patterns of marker combinations that enrich for insulin^+^ cells***.


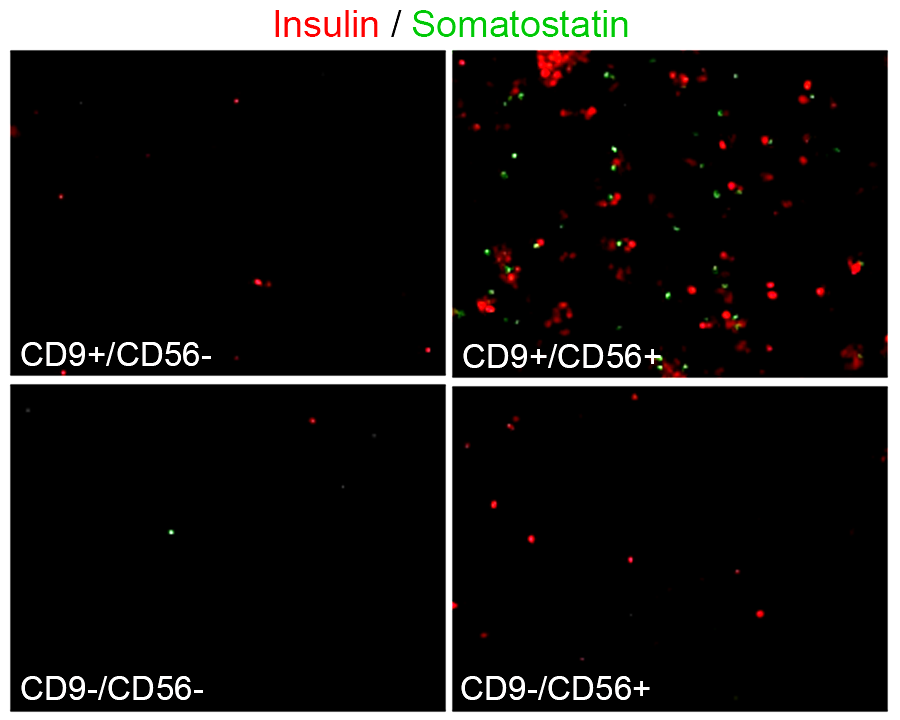


**Figure C: *Enrichment of insulin^+^ and somatostatin^+^ cells in the CD9^+^/CD56^+^ compartment.***

**
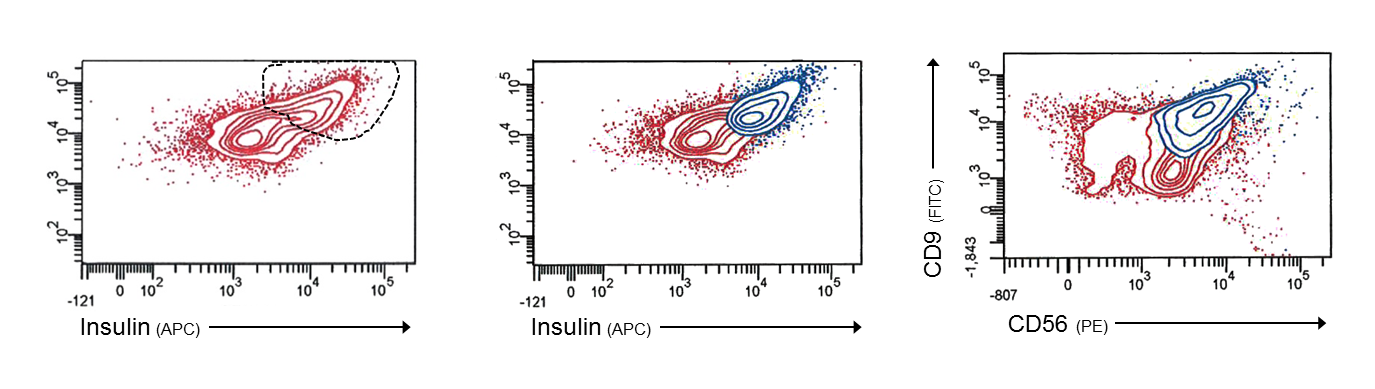
**

Non-specific label

b.

a.

**Figure D: *Determination of insulin^+^ cells.***
