# Supplementary material for: Functional Proteomics Screen Enables Enrichment of Distinct Cell Types from Human Pancreatic Islets
Source: PLoS One. 2015 Feb 23;10(2):e0115100. doi: 10.1371/journal.pone.0115100 (PMC4338300; doi:10.1371/journal.pone.0115100)
Supplement: S2 File — Procedure for combined extracellular and intracellular flow cytometry analysis. (DOC) [file pone.0115100.s002.doc]

**Procedure for combined extracellular and intracellular flow cytometry analysis.**

Islet cell debris and clusters were gated out using FSC/SSC, and dead cells were excluded using PI labeling (in live cell assays). CD9^high^ and CD56^+^ cells were identified separately, and then used for defining a gate for CD56^+^ cells which also express high levels of CD9 (CD9^high^/CD56^+^). This gate was increased and decreased by changing the threshold of expression equally along both the CD9 and CD56 axes. Labeled samples were further analyzed for co-staining with insulin (insulin^+^ cells were defined in an independent plot, and viewed by color coding over the CD9/CD56 plot). Purity of beta cells was determined based on the number of insulin^+^ cells within the CD9/CD56 gate divided by the overall number of cells in this gate. Beta cell yield, in turn, was determined by dividing the number of insulin^+^ cells in the CD9/CD56 gate by the number of cells in the insulin gate.
